# Glasgow Coma Scale and Its Components on Admission: Are They Valuable Prognostic Tools in Acute Mixed Drug Poisoning?

**DOI:** 10.1155/2011/952956

**Published:** 2011-03-28

**Authors:** N. Eizadi Mood, A. M. Sabzghabaee, Gh. Yadegarfar, A. Yaraghi, M. Ramazani Chaleshtori

**Affiliations:** ^1^Poisoning Emeregncy Department, Noor and Ali Asghar Medical Center, Isfahan University of Medical Sciences, Ostandari Avenue, Isfahan 81458-31451, Iran; ^2^Isfahan Clinical Toxicology Research Center, Noor and Ali Asghar Medical Center, Isfahan University of Medical Sciences, Ostandari Avenue, Isfahan 81458-31451, Iran; ^3^Department of Epidemiology and Biostatistics, School of Health, Isfahan University of Medical Sciences, Isfahan 81746-73461, Iran

## Abstract

*Introduction*. The verbal, eye, and motor components of Glasgow coma scale (GCS) may be influenced by poisoned patients' behavior in an attempted suicide. So, the values of admission GCS and its components for outcomes prediction in mixed drugs poisoning were investigated. *Materials and Methods*. A followup study data was performed on patients with mixed drugs poisoning. Outcomes were recorded as without complications and with complications. Discrimination was evaluated by calculating the area under the receiver operating characteristic curves (AUC). *Results*. There was a significant difference between the mean value of each component of GCS as well as the total GCS between patients with and without complication. Discrimination was best for GCS (AUC: 0.933 ± 0.020) and verbal (0.932 ± 0.021), followed by motor (0.911 ± 0.025), then eye (0.89 ± 0.028). *Conclusions*. Admission GCS and its components seem to be valuable in outcome prediction of patients with mixed drug poisoning.

## 1. Introduction

Glasgow coma scale (GCS) first appeared in 1974 in reports by Graham Teasdale and Bryan J. Jennet, both professors of neurosurgery at the University of Glasgow [1]. GCS is now used in several emergency departments as an indicator for the status of the central nervous system regardless of their primary etiology. Poisoning with drugs influences the biochemical substances of the brain and causes brain damage. This may change the level of consciousness as well. The GCS has been performed for outcome and recovery evaluation of patients admitted to an intensive care unit (ICU) following drug overdose [2], for the mental status evaluation of poisoning patients [3], the need for intubation in patients with antidepressant poisoning [4], and for predicting acute and delayed poisoning outcomes [5–10]. All previous studies showed that for the assessment of complication and mortality, the GCS score provides the best indicator in poisoned patients; however, no study were done to show how predictive is the subset of GCS (eye, motor, and verbal). Mixed drugs poisoning (MDP) is one of the most common reasons for admission in the poisoning unit of our emergency department. Since the verbal, eye, and motor components of GCS may be influenced by poisoned patients' behavior in an attempted suicide, this study was designed to evaluate the values of GCS and its components in outcomes prediction of patients with MDP.

## 2. Materials and Methods

This is a prospective followup study which conducted at the Poisoning Emergency Department of Noor University Medical Center, a main referral center of Isfahan Province, Iran. The centre is facilitated, staffed, and designed exclusively for the management of poisoned patients in our hospital. One hundred fifty two patients with mixed drugs poisoning (MDP) were hospitalized in the centre and followed over time to measure the final outcome. Patients transferred from elsewhere and patients admitted after the first 24 hours of ingestion were excluded. Having 152 patients, we would be able to estimate 95% confidence interval for mean GCS scores with 0.4 effect size. The project was approved by the Institutional Ethics Committee of Isfahan University of Medical Sciences (IUMS) (Research Project Number, 386295). 

For all patients, routine biochemical tests were measured, and usual treatments were continued. Trained medical staff prospectively recorded demographic data and clinical readings of patients. The composite GCS, including eye, motor, and verbal components were measured on admission. GCS was determined based on three components: eyes (four = opening spontaneously, three = opening to verbal command, two = opening to pain, and one = no eye opening), verbal (five = oriented, four = disoriented, three = inappropriate words, two = incomprehensible sounds, and one = no verbal response), and motor (six = obeys, five = localizes pain, four = withdrawal, three = abnormal flexion, two = abnormal extension, and one = no motor response) [11]. All other available data including toxic agent, gender, and age were also recorded. The outcomes were followed subsequently based on the chart and categorized as either without complications; or with complications from minor to severe (requiring intubation and ventilatory support). Since two patients died and both of them had complications, they included in the complication group. Data were presented as mean ± SE or *n* (%) where appropriate. Two-tailed paired *t*-test was performed to compare mean scores at the baseline and after the treatment. Logistic regression was applied to calculate odds ratio (OR) with 95% confidence interval (CI) to show how predictive is the subset of GCS (eye, motor, and verbal). For each outcome measure and combination of GCS components, we identified the optimal cutoff point. The area under the curve (AUC) and its standard error were calculated to measure the prognostic information provided by each combination of GCS components. AUCs between 0.7 and 0.8 were classified as “acceptable” and between 0.8 and 0.9 as “excellent” discrimination [12]. Data were analyzed using SPSS version 17.0 (SPSS Inc., Chicago, IL, USA) and MedCalc (MedCalc Software Inc, Mariakerke, Belgium). *P* value less than .05 was considered as statistically significant results.

## 3. Results

In this study, 152 patients with mixed drug poisoning (MDP) were studied. There were more men (*n* = 54) than women (*n* = 98). The mean age was 24.89 ± 0.65 years, ranged from 14 to 56 years. Patients were admitted to the hospital early in the course of ingestion (161.99 ± 6.65 minutes). The most frequently used drugs were benzodiazepines, pain relief medications, and antidepressants drugs (Table 1).

Our findings indicated that 130 patients (85.5%) survived without complications, 20 patients (13.2%) survived with complications, and two patients died (1.3%) (Table 2). The length of hospital stay in 26.3% of patients was more than 24 hours. 

There were significant difference in mean value of each component as well as total GCS between patients with and without complication (*P* < .0001) (Table 3). Patients with complications had less verbal, eye, motor, and GCS scores than others.

For eye, verbal, and motor variables, and the area under ROC curve, sensitivity and specificity at the best cutoff point were determined and compared with GCS. Discrimination was excellent for GCS as well as all components including motor, eye, and verbal. There were no statistically significant differences among all components in terms of area under ROC curve (Table 4). 

Logistic regression results indicated that the chance of complications is 44 times higher for patients with motor score less than five in comparison with normal motor score. It also showed that patients with a verbal score equal or less than three had 90 times the risk of complications compared with those with verbal score four. With respect to eye component, the chance of complications is 52 times higher for patients with eye score less than two in comparison with normal eye score (score ≥ 3) (Table 5).

## 4. Discussion

In this study, the value of GCS and its components in predicting outcome of patients with MDP were investigated. Our findings show the mean value of each component as well as total GCS in patients with MDP was strongly related with outcomes. Patients without complications had greater mean values than patients with complications. The prognosis in patients presenting with poisoning depends on the level of consciousness on admission [13].

Although rapid changes in the level of consciousness in poisoning cases may raise a question on the role of admission GCS in predicting outcome, we showed that admission GCS as well as its components can be validated for poisoned patients with mixed drugs ingestion. Applicability of GCS in outcome prediction of different poisoning has been evaluated previously. GCS has been shown to be an effective clinical parameter that helps clinicians to predict the outcome of organophosphate poisoning cases in the initial assessment [14–17]. GCS has been also used for predicting neuropsychological sequels of carbon monoxide (CO) poisoning [7, 10]. 

Our results showed patients with GCS equal or less than 10 had higher chance of getting complications in comparison with patients with GCS score more than 10. Unverir et al. demonstrated that antidepressant-poisoned patients with GCS scores of 8 or less were intubated more frequently [4]. GCS less than eight had been more associated with mortality in children presenting with poisoning or intoxication [5]. Heyman et al. recommended that patients with a GCS score of more than six, and who are not intubated, may not need admission to an intensive care unit in intentional drug overdose cases [18]. Hamad et al. illustrated the patients with drugs overdose and a GCS score of less than 13 needed admission to an ICU [19]. GCS score equal or less than 14 had been associated with myocardial injury in CO poisoning [10]. Difference in GCS score in different studies may be due to different toxic agent studied, different poisoned patient population, and evaluating different outcomes. Toxic agent [20–22], antidote [23], and the kind of intervention by different physicians [24] are also effective variables at predicting outcome. Davies et al. illustrated besides of GCS, the kind of pesticide affected the outcome in acute organophosphate (OP) poisoning [20]. The pattern of changing GCS score after using antidote for outcome prediction may be another important issue [23]. 

Variability in agreement between physicians when measuring the GCS is another concern that may limit its clinical usefulness [25, 26]. The interobserver variability is high when the scoring systems are not used on a regular basis, thus affecting the accuracy and reproducibility of the data [27, 28]. This is potentially relevant in our study, as GCS determination was performed by several physicians and, before the study period, had not formed a routine part of patient assessment. We tried to minimize variability by having one person to coordinate the process of data collection and, before the study, had our anesthesiologist or toxicologist formally train our emergency physicians in the assessment of GCS. All GCS determinations were determined by this group of physicians.

## 5. Conclusions

In conclusion, our results show admission GCS and its components seem to be valuable prognostic tools in acute mixed drug poisoning. Findings on the predictive ability of GCS score and its components can help clinicians to better identify patients who may develop complications.

There are also some limitations in our study.

1. Our results may not be extrapolated to other institutions. It is a single-centre study, which may not be representative of all patients.
2. GCS measured at the time of admission may not reflect completely unforeseen events that may be major determinants of outcome. A sequential evaluation of GCS may yield greater accuracy [29].
3. The overall number of our patients with complications or death was relatively small which may be affected power of prediction of complications by GCS scores. Given the listed most common agents of poisoning employed by the study population (benzodiazepines, pain relief medications including acetaminophen, etc.), it is likely that mortality and serious complications were negligible. Also, most of the previous studies have only included patients admitted to an intensive care unit. In contrast, our study included all poisoned patients presenting to the poisoning emergency department, which makes the results more generalizable.
4. In our hospital there is not a toxicology lab; therefore, the toxicological screening was not carried out to confirm different kind of toxic agents. The diagnosis was based on history and clinical evaluation and summary records which had been confirmed by our poisoning emergency department specialists. Although “mixed drugs” toxicity has been taken, but any single agent may alter the outcome should also be considered.
5. Lack of measuring intensity of treatment may be affected the rate of complications.

Therefore, we suggest performing a larger prospective study of poisoned patients, comparing complication or death prediction of GCS scores and its components which measured over time.

## Figures and Tables

**Table 1:** List of different medications taken by patients.

Drugs	*N*
Benzodiazepines	96
Pain relief medications*	79
Antidepressants	58
Antihistamines	22
Antihypertensive	20
Opioids	15
Anti-convulsants	14
Hypoglycemic agents	4
Other medications	66

*Pain relief medications including acetaminophen, Adult cold, Non-steroidal anti-inflammatory drugs; *N*: number of patients.

**Table 2:** Outcomes distribution in patients with mixed drug poisoning (MDP).

Outcomes	Number (%)
Without complications	130 (85.5)
With Complications	
Minor	10 (6.6)
Severe	12 (7.9)

**Table 3:** The mean value of GCS and its components in patients with MDP with respect to outcomes.

Variable	Outcomes Groups	Mean ± SE	Mean difference ± SE	95% CI of difference	
				Lower	Upper
Motor	without complication	5.75 ± 0.06	2.01 ± 0.29	1.41	2.62
	complications	3.73 ± 0.28			
Verbal	without complication	4.38 ± 0.07	2.3 ± 0.19	1.95	2.72
	complications	2.05 ± 0.22			
Eye	without complication	3.42 ± 0.06	1.59 ± 0.16	1.26	1.93
	complications	1.82 ± 0.19			
GCS	without complication	13.56 ± 0.17	5.97 ± 0.46	5.05	6.89
	complications	7.59 ± 0.52			

MDP: mixed drug poisoning; SE: standard error of mean; CI: Confidence Interval; *N*: number of patients.

**Table 4:** Areas under the ROC curves of each component as well as total GCS.

Component	AUC ± SE (95% CI)	Cutoff point	Sensitivity (95% CI)	Specificity (95% CI)
Motor	0.911 ± 0.025 (0.85–0.95)	equal or less than 5	90.91 (70.8–98.6)	81.54 (73.8–87.8)
Verbal	0.932 ± 0.021 (0.87–0.96)	equal or less than 3	90.91 (70.8–98.6)	90.00 (83.5–94.6)
Eye	0.89 ± 0.028 (0.83–0.93)	equal or less than 2	86.36 (65.1–96.9)	89.23 (82.6–94.0)
GCS	0.933 ± 0.020 (0.88–0.96)	equal or less than 10	90.91 (70.8–98.6)	92.31 (86.3–96.2)

ROC: Receiver under Operating Curve; GCS: Glasgow Coma Scale; AUC: area under the curve; SE: Standard error of mean; CI: Confidence Interval.

**Table 5:** Prediction of outcomes in patients with MDP.

Variable	*N*	*B*	SE	*P* value	Unadjusted OR (95% CI)	Adjusted OR* (95% CI)
Motor score						
6	108				1	1
≤5	44	3.79	0.77	.000	44.16 (9.66–20.185)	41.16 (8.67–19.54)
Verbal Score						
4 and 5	119				1	1
≤3	33	4.50	0.79	.000	90 (18.86–429.3)	153.26 (23.79–987.2)
Eye Score						
3 and 4	119				1	1
≤2	33	3.96	0.68	.000	52.47 (13.76–200)	51.14 (12.59–207.7)
GCS Score						
11–15	122				1	1
≤10	30	4.78	0.81	.000	120 (24.46–588)	198 (28.63–1370)

*The OR was adjusted for age and gender.

MDP: mixed drug poisoning; *N*: Number of the patients; *B*: estimated coefficient; SE: standard error of mean; OR: odds ratio; CI: Confidence Interval; GCS: Glasgow coma scale.
